# Bronchoalveolar Lavage Fluid Cytomegalovirus DNA Load as a Predictor of Mortality in AIDS Patients with Pulmonary Infections: A Retrospective Cohort Study

**DOI:** 10.3390/pathogens15040377

**Published:** 2026-04-01

**Authors:** Junyang Yang, Min Zhang, Renfang Zhang, Jun Chen, Yinzhong Shen, Tangkai Qi, Zhenyan Wang, Wei Song, Yang Tang, Jianjun Sun, Shuibao Xu, Youming Chen, Yueming Shao, Li Liu, Hongzhou Lu

**Affiliations:** 1 Department of Infectious and Immune Diseases, Shanghai Public Health Clinical Center, Fudan University, Shanghai 201508, China; yangjunyang@shaphc.org (J.Y.); zhangrenfang@shaphc.org (R.Z.); chenjun@shaphc.org (J.C.); shenyinzhong@shaphc.org (Y.S.); qitangkai@shaphc.org (T.Q.); wangzhenyan@shaphc.org (Z.W.); songwei@shaphc.org (W.S.); tangyang@shaphc.org (Y.T.); sunjianjun@shaphc.org (J.S.); xushuibao@shaphc.org (S.X.); chenyouming@shaphc.org (Y.C.); shaoyueming@shaphc.org (Y.S.); 2 Medical Laboratory, Shanghai Public Health Clinical Center, Fudan University, Shanghai 201508, China; zhangmin30133@shaphc.org; 3 National Clinical Research Center for Infectious Diseases, Shenzhen Third People’s Hospital, The Second Affiliated Hospital of Southern University of Science and Technology, Shenzhen 518112, China

**Keywords:** cytomegalovirus, HIV/AIDS, bronchoalveolar lavage fluid, viral load, mortality, pneumonia

## Abstract

Cytomegalovirus (CMV) pneumonia presents diagnostic challenges in AIDS patients, as plasma monitoring often fails to reflect pulmonary viral burden. This retrospective study evaluated the prognostic value of bronchoalveolar lavage fluid (BALF) CMV DNA loads in 189 AIDS patients with pulmonary infections and CD4^+^ T cell counts < 200 cells/μL. CMV DNA in BALF and plasma was quantified to analyze associations with immune status and 90-day all-cause mortality. CMV detection was significantly more frequent in BALF (49.7%) than plasma (26.6%), indicating viral compartmentalization. An optimal BALF cutoff of 10,000 copies/mL was established for mortality prediction. Patients exceeding this threshold exhibited significantly lower CD4^+^ counts, increased mechanical ventilation requirements (34.4% vs. 11.5%), and prolonged hospital stays. Crucially, a BALF CMV load > 10,000 copies/mL was identified as an independent predictor of 90-day mortality (adjusted odds ratio = 3.78; 95% CI: 1.12–12.71). In conclusion, pulmonary CMV replication is prevalent and often compartmentalized in AIDS patients. A BALF CMV DNA load exceeding 10,000 copies/mL serves as a biomarker of profound immunosuppression and independently predicts poor clinical outcomes, highlighting the necessity of quantitative BALF monitoring for risk stratification.

## 1. Introduction

Cytomegalovirus (CMV), a pervasive opportunistic pathogen, poses significant challenges in immunocompromised cohorts, notably affecting 10–40% of allogeneic hematopoietic stem cell transplant (allo-HSCT) recipients, with pneumonia as the most severe manifestation, showing mortality rates up to 70% [1]. Diagnostic criteria for CMV pneumonia have evolved significantly since the 1993 consensus, categorizing cases into confirmed—requiring viral isolation from lung tissues—and clinical, based on bronchoalveolar lavage fluid (BALF) viral presence combined with respiratory symptoms, although standardized thresholds remain contentious [2].

In Acquired Immunodeficiency Syndrome (AIDS) patients, confirmed CMV pneumonia is seldom reported, largely via case studies [3,4,5]. In contrast, Goussard et al. reported that among *Human Immunodeficiency Virus* (*HIV*)-infected infants with suspected *Pneumocystis jirovecii* pneumonia (PCP) unresponsive to trimethoprim–sulfamethoxazole (TMP-SMX) therapy, lung biopsy revealed CMV pneumonia in 72% of cases [6]. This high diagnostic yield suggests that CMV pneumonia is frequently overlooked in AIDS patients, likely due to its nonspecific presentation (fever, cough, dyspnea, diffuse infiltrates on imaging) that mimics PCP and other pulmonary infections, combined with the impracticality of confirmatory lung biopsies.

Advances in molecular detection have identified CMV DNA in BALF from people living with HIV (PLWH), suggesting higher-than-expected CMV pneumonia prevalence [7,8,9,10]. While BALF CMV DNA load correlates with pneumonia risk in non-HIV immunocompromised patients, diagnostic thresholds vary considerably due to heterogeneity in immune status and co-infections [11,12,13,14]. In PLWH, this is further complicated: CD4^+^ T cell depletion may enhance viral replication, yet intact pulmonary innate immunity could limit pathogenicity. Whether co-infection-induced inflammation (e.g., PCP, tuberculosis) affects CMV load dynamics remains undefined.

This study investigates the clinical significance of BALF CMV DNA viral load in AIDS patients with pulmonary infections, aiming to establish PLWH-specific diagnostic thresholds and define their relationships with co-infections, immune status, and prognosis, thereby providing evidence-based guidance for clinical management.

## 2. Materials and Methods

### 2.1. Study Population

This retrospective observational cohort study was conducted at the Shanghai Public Health Clinical Center. We systematically reviewed medical records of HIV/AIDS patients hospitalized between July 2022 and December 2024. The inclusion criteria were as follows: ① confirmed HIV-positive status; ② chest computed tomography (CT) imaging showing infectious pulmonary lesions; ③ CD4^+^ T cell below 200 cells/μL; ④ age 18 years or older; and ⑤ bronchoscopy with bronchoalveolar lavage (BAL) performed within 7 days of hospital admission. For patients who underwent multiple BAL procedures during a single episode of pulmonary infection, only the first specimen was included to avoid duplicate data. Exclusion criteria included: ① pregnant or lactating women; ②loss to follow-up resulting in unknown 90-day survival status; and ③concurrent autoimmune diseases or other conditions requiring systemic immunomodulatory treatment, such as solid organ transplantation, or long-term high-dose corticosteroid therapy.

### 2.2. Clinical Procedures and Data Collection

Bronchoscopy and BAL were performed in accordance with standard clinical protocols at our center. Generally, BAL was conducted by instilling sterile saline into the affected lung segment identified on CT imaging. BALF samples were collected for diagnostic purposes based on the attending physician’s judgment. Paired plasma CMV DNA data were included if the blood sample was collected within 48 h of the bronchoscopy procedure. Patients without concurrent plasma CMV testing were excluded from the plasma–BALF correlation analysis.

We collected patient demographic and disease-related data from the hospital’s electronic medical records system. Demographic information included sex and age. Disease-related data encompassed antiretroviral therapy (ART) status, oxygen support requirements, comorbidities, prognosis, length of hospital stay, and pertinent laboratory tests. Patient survival status was documented at hospital discharge. For patients discharged alive, telephone follow-up was performed to ascertain 90-day survival status.

### 2.3. Laboratory Measurements

As this was a retrospective study based on real-world clinical practice, BALF samples were not archived but were transported immediately to the Department of Clinical Laboratory upon collection. CMV DNA viral loads in both fresh BALF and plasma were quantified using a commercial real-time polymerase chain reaction (PCR) assay (Human Cytomegalovirus Nucleic Acid Detection Kit, Sansure Biotech Inc., Changsha, China) on an ABI 7500 system (Applied Biosystems, Thermo Fisher Scientific, Waltham, MA, USA). Because this is a proprietary commercial in vitro diagnostic kit, the exact sequences of the primers and fluorescent probes are protected as intellectual property by the manufacturer and are not disclosed.

Briefly, sample preparation was performed according to the manufacturer’s instructions: 1 mL of the raw BALF sample was centrifuged at 12,000 rpm for 5 min. After discarding the supernatant, the pellet was resuspended and lysed in 50 μL of nucleic acid release reagent. Subsequently, 10 μL of the pre-treated sample was added to a PCR tube containing 40 μL of the master mix (incorporating reaction buffer, enzymes, primers, probes, and an internal control). The thermal cycling conditions consisted of an initial incubation at 50 °C for 120 s and denaturation at 94 °C for 300 s, followed by 45 cycles of amplification (94 °C for 15 s and 57 °C for 30 s), and a final cooling step at 25 °C for 10 s.

In standard clinical diagnostic workflows, the viral load is calculated based on standard curves and reported volumetrically (copies/mL of the original fluid). Therefore, human cell quantification in BALF was not performed for viral load normalization. The lower limit of quantification for this assay was 400 copies/mL.

CD4^+^ T cell counts were measured by flow cytometry. Diagnosis of pulmonary co-infections (e.g., PCP, mycobacteria, fungi) was based on the attending physician’s comprehensive judgment combined with established microbiological criteria using smears, cultures, or molecular assays from respiratory specimens.

### 2.4. Data Analysis

All statistical analyses were performed using SPSS version 26.0 (IBM Corp., Armonk, NY, USA), and figures were generated using GraphPad Prism version 8.0 (GraphPad Software, San Diego, CA, USA). Continuous variables were first assessed for normality using the Kolmogorov–Smirnov test. Normally distributed variables were expressed as mean ± standard deviation (SD), with comparisons between two groups conducted using independent samples *t*-tests. Non-normally distributed variables were expressed as median and interquartile range (IQR), with comparisons between two groups conducted using Mann–Whitney U tests. Comparisons among multiple groups (e.g., different co-infection etiologies) were performed using the Kruskal–Wallis H test. Categorical variables were presented as frequencies and percentages, with differences evaluated using χ^2^ tests or Fisher’s exact test as appropriate. Correlations between BALF CMV DNA viral load and immunological or virological parameters (including CD4^+^ T cell count, CD4^+^/CD8^+^ ratio, HIV RNA levels, and plasma CMV DNA levels) were assessed using Spearman rank correlation analysis. The diagnostic performance of BALF CMV DNA viral load for predicting 90-day all-cause mortality was evaluated using receiver operating characteristic (ROC) curve analysis. The optimal cutoff value was determined using the Youden index (maximum [sensitivity + specificity − 1]).

Univariate logistic regression analysis was performed to identify potential risk factors associated with 90-day mortality. Variables with *p* < 0.10 in the univariate analysis were subsequently included in the multivariate logistic regression model to determine independent predictors. Results were presented as odds ratios (OR) and adjusted odds ratios (aOR) with their 95% confidence intervals (CIs). Survival analysis employed the Kaplan–Meier method to construct survival curves, with differences tested using the Log-rank test. All statistical tests were two-sided, with statistical significance defined as *p* < 0.05.

## 3. Results

### 3.1. Baseline Characteristics

A total of 189 AIDS patients with pneumonia were enrolled in this study. The cohort, predominantly comprising ART-naïve males, exhibited profound immunosuppression as evidenced by severe CD4^+^ T cell depletion and high HIV viral loads. Crucially, a significant discordance was observed in CMV compartmentalization: CMV DNA detection was significantly more frequent in BALF compared to plasma (49.74% vs. 26.62%, *p* < 0.05). Furthermore, high-level viral loads (>10,000 copies/mL) were predominantly a feature of the lung compartment (16.93%), whereas high-level viremia in plasma was rare (1.44%, *p* < 0.001). Detailed baseline characteristics are summarized in Table 1.

### 3.2. Similar BALF CMV Burden Across Pulmonary Co-Infections

**Figure 1:**
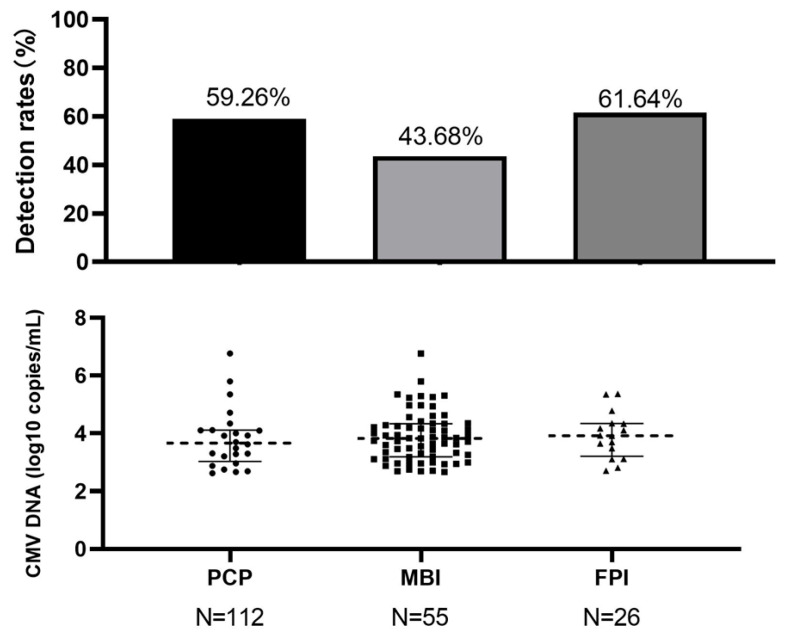
BALF CMV DNA detection rates and CMV DNA viral loads among patients with different types of pulmonary infections.

We analyzed CMV compartmentalization across different co-infection etiologies, including PCP, mycobacteria, and fungi. Analysis revealed no statistically significant differences in either BALF CMV DNA detection rates (*p* = 0.13) or median viral loads (*p* = 0.59) among these pathogen groups (Figure 1).

### 3.3. Correlation Between Immunological or Virological Indicators and BALF CMV DNA Levels

**Figure 2:**
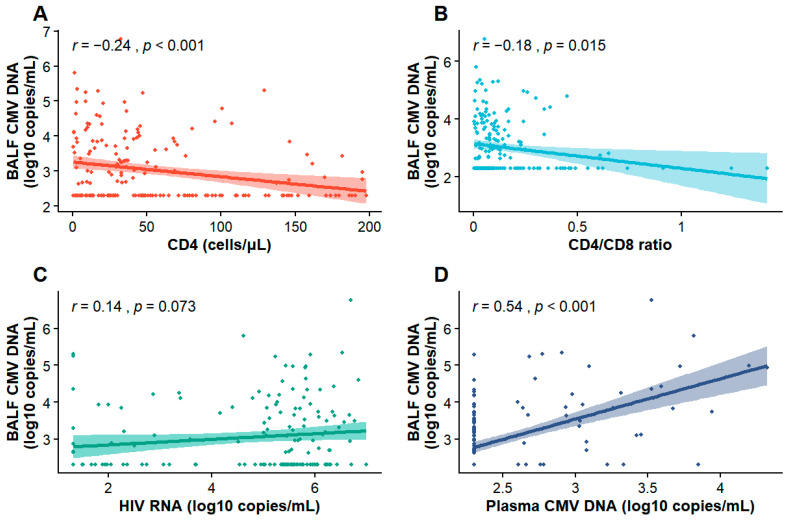
Correlation analyses between BALF CMV DNA levels and selected immunological and virological parameters. The scatter plots depict the associations between BALF CMV DNA levels and CD4^+^ T cell (**A**), CD4/CD8 ratio (**B**), HIV RNA levels (**C**), and plasma CMV DNA levels (**D**).

To explore factors associated with CMV replication in the lower respiratory tract, we assessed the correlations between immunological or virological parameters and CMV DNA levels in BALF. In terms of immunological markers, CD4^+^ T cell counts exhibited a weak inverse correlation with BALF CMV DNA levels (r = −0.24, *p* < 0.001) (Figure 2A), and a similar weak negative correlation was observed for the CD4/CD8 ratio (r = −0.178, *p* = 0.01) (Figure 2B). Regarding virological indicators, no significant correlation was observed between plasma HIV RNA levels and BALF CMV DNA levels (r = 0.141, *p* = 0.0729) (Figure 2C). However, plasma CMV DNA levels showed a moderate positive correlation with BALF CMV DNA levels (r = 0.538, *p* < 0.001) (Figure 2D).

### 3.4. High BALF CMV DNA Viral Load Is an Independent Predictor of Mortality

In the univariate analysis (Table 2), several factors were associated with patient mortality. Specifically, a high CMV DNA load in the BALF (>10,000 copies/mL) was significantly associated with an increased risk of death (OR = 3.05, 95% CI: 1.01–9.22, *p* = 0.04). No significant associations were observed for lower viral loads (400–2000 copies/mL: OR = 0.67, *p* = 0.61; 2001–10,000 copies/mL: OR = 1.74, *p* = 0.40). Additionally, elevated total bilirubin (TBIL) (per 1 μmol/L increase: OR = 1.07, 95% CI: 1.01–1.13, *p* = 0.02), procalcitonin (PCT) (per 1 ng/mL increase: OR = 1.20, 95% CI: 1.05–1.38, *p* < 0.01), and C-reactive protein (CRP) (per 1 mg/L increase: OR = 1.01, 95% CI: 1.00–1.01, *p* = 0.02) were associated with poor outcomes. Conversely, higher hemoglobin (Hb) levels were protective (per 10 g/L increase: OR = 0.82, 95% CI: 0.67–0.99, *p* = 0.05). Other clinical variables, including age, CD4^+^ T-cell count (<50 vs. ≥50 cells/μL), HIV RNA load, and ART status, were not significantly associated with survival.

In the multivariate analysis (Table 2), BALF CMV DNA load > 10,000 copies/mL remained the only significant independent predictor of mortality, with the risk of death increasing nearly four-fold compared to the reference group (aOR = 3.78, 95% CI: 1.12–12.71, *p* = 0.03). The associations for TBIL (*p* = 0.24), Hb (*p* = 0.39), PCT (*p* = 0.29), and CRP (*p* = 0.46) were no longer significant in the multivariate analysis.

### 3.5. Predictive Value and Threshold Determination for BALF CMV DNA

**Figure 3:**
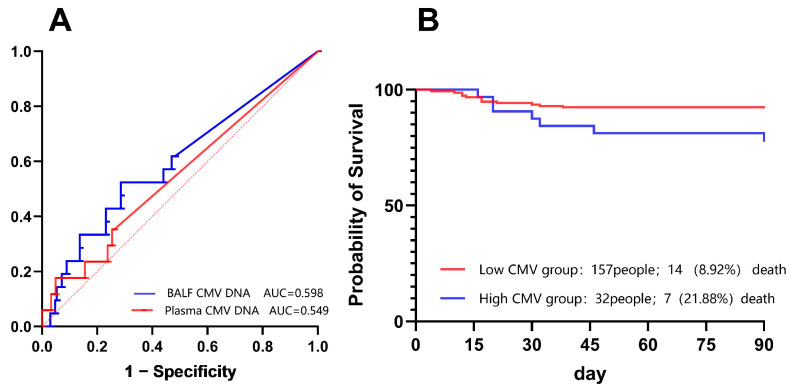
Performance of CMV viral loads in predicting clinical outcomes and survival analysis. (**A**) ROC curves comparing the predictive value of BALF CMV DNA versus plasma CMV DNA for 90-day all-cause mortality. The dotted diagonal line represents the reference line of no discrimination (AUC = 0.5). (**B**) Kaplan–Meier survival curves for 90-day mortality stratified by different BALF CMV DNA viral load levels. Abbreviation: BALF, bronchoalveolar lavage fluid.

We performed ROC analysis to evaluate the prognostic utility of BALF CMV DNA loads. Regarding 90-day all-cause mortality (Figure 3A), BALF CMV DNA demonstrated a modest discriminative ability with an area under the curve (AUC) of 0.598 (95% CI: 0.462–0.733), which was marginally higher than that of plasma CMV DNA (AUC = 0.549; 95% CI: 0.396–0.701).

While the overall sensitivity was limited, the analysis revealed that elevated BALF viral loads possessed substantial specificity for predicting mortality. Specifically, thresholds ranging from 7455 to 12,850 copies/mL achieved specificities between 81.6% and 86.3%. Prioritizing specificity to identify patients at the highest risk, and considering clinical practicality, we established a rounded cutoff of 10,000 copies/mL. This threshold was subsequently used to stratify patients into high- and low-viral load groups for survival analyses.

### 3.6. Clinical and Immunological Characterization of High vs. Low CMV Groups

Stratification of the cohort based on the cutoff of 10,000 copies/mL identified 32 patients (16.9%) in the High CMV Group and 157 patients (83.1%) in the Low CMV Group. While demographic baselines such as age and sex were comparable between the groups, elevated BALF CMV DNA levels served as a distinct marker of profound immunosuppression and disease severity.

Patients in the High CMV Group exhibited significantly lower CD4^+^ T cell counts [median 19.1 vs. 40.7 cells/μL, *p* < 0.01] and reduced serum albumin levels (*p* = 0.01). Consistent with this severe immune depletion, fewer patients in the High CMV Group were receiving ART at admission (18.8% vs. 43.3%, *p* < 0.01). The burden of opportunistic co-infections was also markedly elevated in this group; specifically, the prevalence of PCP reached 81.3%, significantly higher than the 54.8% observed in the Low CMV Group (*p* < 0.01).

Clinically, the High CMV Group phenotype translated into poorer outcomes. Patients in this group had a significantly increased requirement for mechanical ventilation (34.4% vs. 11.5%, *p* < 0.01) and endured prolonged hospitalization (*p* < 0.001). Most importantly, survival analysis highlighted the lethal impact of high-level CMV replication. Although the crude mortality comparison showed a borderline trend (Table 3), Kaplan–Meier analysis revealed a significantly lower 90-day survival probability in the High CMV Group compared to the Low CMV Group (Log-rank test, *p* = 0.037; Figure 3B). The hazard ratio indicated that patients with high BALF CMV DNA loads faced a 3.4-fold increased risk of death (Mantel-Haenszel HR = 3.41, 95% CI: 1.07–10.81).

### 3.7. Impact of Anti-CMV Therapy on Prognosis

To further evaluate the combined impact of baseline CMV viral load and anti-CMV therapy on patient prognosis, the cohort was stratified into four subgroups: Low CMV/Untreated (*n* = 134), Low CMV/Treated (*n* = 23), High CMV/Untreated *n* = 19), and High CMV/Treated (*n* = 13). As shown in Table 4, the 90-day mortality rates for these groups were 8.21% (11/134), 13.04% (3/23), 21.05% (4/19), and 23.07% (3/13), respectively. Compared to the Low CMV/Untreated reference group, patients with a high CMV load and those receiving antiviral therapy exhibited notably higher mortality rates. However, likely due to the limited sample sizes within the treatment and high viral load subgroups, these differences did not reach statistical significance using Fisher’s exact test (Low CMV/Treated, *p* = 0.434; High CMV/Untreated, *p* = 0.095; High CMV/Treated, *p* = 0.111).

## 4. Discussion

This study reveals the underestimated burden and prognostic significance of pulmonary CMV replication in PLWH. CMV DNA was detected in the BALF of 49.7% of patients—nearly double the rate in plasma (26.6%)—with 16.9% exceeding a high-load threshold of 10,000 copies/mL. This high viral load emerged as a robust independent predictor of 90-day mortality (aOR = 3.78) and correlated with increased respiratory support requirements and prolonged hospitalization. These data challenge historical paradigms framing CMV in PLWH primarily as a retinal or gastrointestinal pathogen [15,16]. The marked compartmentalization of viral replication implies that plasma monitoring systematically underestimates pulmonary CMV burden. This parallels recent molecular evidence revealing frequent, clinically overlooked CMV coinfection in PLWH [17,18,19].

Historically, the prognostic relevance of pulmonary CMV in advanced HIV has been recognized, yet diagnostic limitations have hampered its clinical application. Foundational research in the 1990s established pulmonary CMV as a robust negative prognostic indicator. For instance, Hayner et al. demonstrated that isolating CMV from BAL fluid indicated a poor prognosis, with 3- and 6-month mortality rates escalating to 28% and 47%, respectively—markedly exceeding the 10% and 15% observed in CMV-negative cohorts [20]. Expanding on these findings, Uberti-Foppa et al. highlighted that positive BAL cultures foreshadowed severe systemic dissemination, given the significantly higher incidence of subsequent extrapulmonary CMV disease in these patients (27% vs. 7%) [21]. However, these early studies were fundamentally limited by their reliance on qualitative viral culture. The diagnostic paradigm shifted toward quantification when Spector et al. demonstrated that plasma CMV DNA burden served as an independent, load-dependent predictor of survival, conferring a 2.5-fold increased mortality risk [22]. Despite these milestones confirming the systemic prognostic value of viral load, the quantitative thresholds required to assess CMV burden and predict outcomes within BALF have remained incompletely defined for PLWH.

Our study directly addresses this long-standing clinical gap. Interpreting BALF CMV viral loads remains complex, primarily given the ubiquity of asymptomatic CMV shedding in immunocompromised hosts. This diagnostic uncertainty is exacerbated in PLWH, who frequently present with polymicrobial pulmonary coinfections [23], complicating the attribution of pathology solely to CMV. While definitive histopathology is required to validate positive controls, lung tissue biopsy is clinically impractical in unstable patients [24]. Specific viral load cutoffs thus offer a vital surrogate for tissue diagnosis. Karoline et al. previously suggested that BALF loads exceeding 10,000 copies/mL indicate substantial viral replication within the lung parenchyma [25]. By demonstrating that a BALF load > 10,000 copies/mL effectively serves as a specific “rule-in” marker for high mortality risk (specificity >80%, aOR = 3.78), our study corroborates this biologically relevant threshold with robust prognostic evidence, providing a pulmonary equivalent to the plasma load model established by Spector et al. [22]. Although our ROC analysis yielded a modest overall discriminatory power (AUC = 0.598)—likely reflecting the multifactorial nature of AIDS-related mortality—this quantitative threshold strongly challenges the older hypothesis that pulmonary CMV functions merely as an “innocent bystander” [26,27,28]. Instead, our observation that patients with high BALF CMV loads experienced significantly elevated mortality and ventilator dependence supports recent evidence characterizing CMV as a deleterious synergistic co-pathogen [15,27,28,29,30,31]. For instance, Jensen et al. reported that among PCP patients receiving corticosteroid therapy, those with BALF CMV isolation exhibited twice the 3-month mortality of CMV-negative patients [32].

A critical unresolved question is whether detecting high BALF CMV DNA mandates specific antiviral therapy. In the context of solid organ transplant (SOT) and allo-HSCT, the clinical pathway is well-defined: targeted anti-CMV therapy is standard of care not only for patients with suspected CMV pneumonia but also for those with asymptomatic viremia (preemptive therapy). This aggressive approach has been proven to significantly alleviate clinical symptoms, prevent progression to end-organ disease, and reduce all-cause mortality [1,33,34]. However, in PLWH, the benefit remains ambiguous. To explore this, we stratified our cohort by viral load and treatment status (Table 4). It is noteworthy that patients receiving anti-CMV therapy exhibited slightly higher mortality rates than their untreated counterparts within the same viral load strata (e.g., 23.07% vs. 21.05% in the high CMV groups; 13.04% vs. 8.21% in the low CMV groups). Although these differences did not reach statistical significance (*p* > 0.05), this counterintuitive trend is a classic reflection of “confounding by indication” in real-world clinical practice. In our cohort, anti-CMV therapy was typically reserved for the most critically ill individuals or those presenting with definitive CMV end-organ diseases, meaning the treated subgroups were inherently sicker and had a poorer baseline prognosis. Furthermore, the routine administration of traditional anti-CMV agents (e.g., ganciclovir) carries significant risks, particularly myelosuppression, which can further complicate the profound cytopenias already present in advanced AIDS [22,35]. Finally, the extremely small sample sizes in the treated subgroups significantly limited our statistical power to detect a true difference (Type II error). Instead of solely relying on immune reconstitution, we propose that high BALF CMV load serves as a marker of profound systemic immunodeficiency, as evidenced by the strong inverse correlation with CD4^+^ T cell counts in our data. While rapid initiation of effective ART remains the cornerstone of management, determining whether specific CMV intervention offers additional benefit is crucial. Therefore, prospective randomized controlled trials (RCTs) are urgently needed to establish the optimal therapeutic strategy. Specifically, future studies should prioritize evaluating the efficacy and safety of newer, lower-toxicity agents like letermovir in this population. Unlike ganciclovir, letermovir does not cause myelosuppression [36], making it a highly promising candidate for RCTs aiming to balance viral suppression with safety in fragile PLWH.

This study has several limitations. First, its retrospective design introduces inherent selection biases and prevents the establishment of causality. For instance, treatment decisions were not randomized, leading to potential confounding by indication that limits any definitive conclusions about therapeutic efficacy. Second, comprehensive CMV diagnostic workups, including serology (IgM/IgG), pp65 antigenemia, and urine testing, were not routinely performed. Although limited available data (100% IgG-positive in 36 tested patients) suggests the detected BALF CMV primarily represents viral reactivation rather than acute primary infection, distinguishing true invasive CMV pneumonia from high-burden viral colonization based solely on BALF PCR remains inherently controversial. Therefore, a high BALF CMV load in this context should be interpreted cautiously as a prognostic biomarker reflecting profound local immune exhaustion, rather than a definitive histological diagnosis of invasive disease. Third, as a single-center study, our findings may have limited generalizability. The patient cohort, likely representing a population with severe disease requiring bronchoscopy, may not be fully representative of the broader PLWH population, potentially affecting the external validity of our proposed viral load threshold. Fourth, our quantitative assay was not calibrated against the World Health Organization (WHO) international standard [37], which restricts the direct transferability of the 10,000 copies/mL threshold to other laboratories using different assays. Finally, the absence of longitudinal viral load monitoring precluded any assessment of CMV replication dynamics or the virological response to therapy.

In conclusion, BALF CMV DNA detection is highly prevalent but frequently overlooked in AIDS-related pulmonary infections. A viral load exceeding 10,000 copies/mL serves as a critical biomarker, independently predicting mortality, mechanical ventilation needs, and prolonged hospital stays. Quantitative BALF CMV monitoring is essential for identifying high-risk PLWH and guiding integrated management strategies centered on immune reconstitution.

## Figures and Tables

**Table 1:** Patient Demographic and Clinical Data.

Item	*n* = 189
Sex, *n* (%):	
Male	175 (92.59%)
Female	14 (7.41%)
Age (years)	46.00 (35.50–49.00)
HBV, *n* (%)	8 (4.23%)
ART, *n* (%)	74 (39.15%)
aCCI score	
Non-infectious comorbidities, *n* (%)	7.00 (6.00–9.00)
Malignancies	15 (7.94%)
Diabetes mellitus	10 (5.29%)
Cardiovascular diseases	7 (3.70%)
Cerebrovascular diseases	2 (1.06%)
Others	6 (3.17%)
Oxygen Support, *n* (%):	
No requirement	44 (23.28%)
Mechanical ventilation	29 (15.35%)
CD4^+^ T Cell Count (cell/μL)	35.83 (13.37–80.60)
CD8^+^ T Cell Count (cell/μL)	355.30 (208.80–637.30)
CD4^+^/CD8^+^ Ratio	0.09 (0.04–0.17)
HIV RNA (log10 copies/mL)	5.53 (4.95–5.99)
BALF CMV DNA, *n* (%)	
<400 copies/mL	95 (50.26%)
400–2000 copies/mL	33 (17.46%)
2000–10,000 copies/mL	29 (15.34%)
>10,000 copies/mL	32 (16.93%)
Plasma CMV DNA (*n* = 139), *n* (%)	
<400 copies/mL	101 (71.94%)
400–2000 copies/mL	23 (16.55%)
2000–10,000 copies/mL	12 (8.63%)
>10,000 copies/mL	2 (1.44%)
WBC (10^9^/L)	5.17 (3.63–7.39)
NEUT% (%)	75.40 (65.35–84.35)
Hb (g/L)	112.80 ± 22.74
PLT (10^9^/L)	219.50 ± 103.00
CRP (mg/L)	40.73 (11.50–74.74)
PCT (ng/mL)	0.10 (0.05–0.36)
ALT (U/L)	20.00 (14.00–35.00)
AST (U/L)	28.00 (20.00–42.00)
ALB (g/L)	32.92 ± 6.42
Globulin (g/L)	34.17 ± 7.33
eGFR (mL/min/1.73 m^2^)	122.00 (97.72–146.5)
Opportunistic Co-infections:	
PCP, *n* (%)	112 (59.26%)
Mycobacterium, *n* (%)	55 (29.10%)
FPI, *n* (%)	26 (13.76%)
Clinical Outcomes:	
90-day all-cause mortality, *n* (%)	21 (11.11%)
Length of hospital stay (days)	18.00 (12.00–29.00)

Abbreviations: ART, antiretroviral therapy; HBV, hepatitis B virus; HIV, human immunodeficiency virus; CMV, human cytomegalovirus; WBC, white blood cell count; NEUT%, neutrophil percentage; Hb, hemoglobin; PLT, platelet count; CRP, C-reactive protein; PCT, procalcitonin; ALT, alanine aminotransferase; AST, aspartate aminotransferase; ALB, albumin; eGFR, estimated glomerular filtration rate; PCP, *Pneumocystis jirovecii* pneumonia; *Mycobacterium*, including pulmonary tuberculosis and *nontuberculous mycobacterial* infections; FPI, fungal pulmonary infections, including aspergillosis, *Talaromyces marneffei* infection, and cryptococcosis; aCCI, age-adjusted Charlson Comorbidity Index; other included peptic ulcer disease/gastrointestinal bleeding (*n* = 2), decompensated liver cirrhosis (*n* = 1), chronic obstructive pulmonary disease (*n* = 1), renal insufficiency (*n* = 1), and hypothyroidism (*n* = 1).

**Table 2:** Logistic regression analysis of factors associated with 90-day mortality.

Variables	Univariate		Multivariate	
	*p* Value	OR (95% CI)	*p* Value	aOR (95% CI)
Male	1.00			
Age (per 1-year increase)	0.74	1.01 (0.97, 1.04)		
aCCI (per 1 increase)	0.23	1.20 (0.89, 1.60)		
BALF CMV DNA (copies/mL)				
<400	Ref	Ref	Ref	Ref
400–2000	0.67	0.70 (0.14, 3.49)	0.68	0.70 (0.13, 3.61)
2001–10,000	0.40	1.74 (0.48, 6.26)	0.53	1.61 (0.37, 7.06)
>10,000	**0.04**	**3.05 (1.01, 9.22)**	**0.03**	**3.78 (1.12, 12.71)**
CD4^+^ T cell counts				
≥50 cell/μL	Ref	Ref		
<50 cell/μL	0.43	0.67 (0.25, 1.81)		
HIV RNA (per log10 increase)	0.61	1.12 (0.73–1.70)		
ART experienced	0.92	0.95 (0.37, 2.42)		
PCP coinfection	0.83	0.91 (0.36, 2.27)		
ALT (U/L)	0.79	1.00 (0.98, 1.01)		
AST (U/L)	0.11	1.01 (1.00, 1.01)		
TBIL (per 1 μmol/L increase)	**0.02**	**1.07 (1.01, 1.13)**	0.24	1.04 (0.97, 1.11)
WBC (per 1 × 10^9^/L increase)	0.28	1.04 (0.97, 1.12)		
Hb (per 10 g/L increase)	**0.05**	**0.82 (0.67, 0.99)**	0.39	0.99 (0.97, 1.01)
PCT (per 1 ng/mL increase)	**<0.01**	**1.20 (1.05, 1.38)**	0.29	1.12 (0.91, 1.39)
CRP (per 1 mg/L increase)	**0.02**	**1.01 (1.00, 1.01)**	0.46	1.00 (1.00, 1.01)

Abbreviations: OR, odds ratio; aOR, adjusted odds ratio; CI, confidence interval; aCCI, age-adjusted Charlson Comorbidity Index; BALF, bronchoalveolar lavage fluid; CMV, cytomegalovirus; ART, antiretroviral therapy; PCP, *Pneumocystis jirovecii pneumonia*; ALT, alanine aminotransferase; AST, aspartate aminotransferase; TBIL, total bilirubin; WBC, white blood cell count; Hb, hemoglobin; PCT, procalcitonin; CRP, C-reactive protein. Note: Variables with *p* < 0.10 in the univariate analysis were included in the multivariate model. Significant *p* values (<0.05) are indicated in bold.

**Table 3:** Demographic, Clinical Characteristics, and Outcomes of Patients Stratified by BALF CMV DNA Load.

Characteristic	Low CMV Group (*n* = 157)	High CMV Group (*n* = 32)	*p* Value
Male sex, *n* (%)	146 (92.99)	29 (90.63)	0.71
Age (years)	46.00 (34.00–58.50)	46.00 (37.25–59.75)	0.70
HBV co-infection, *n* (%)	4 (2.55)	2 (6.25)	0.27
ART experience, *n* (%)	68 (43.31)	6 (18.75)	<0.01
CD4^+^ T cell count (cells/μL)	40.66 (15.87–90.46)	19.07 (8.64–44.89)	<0.01
CD8^+^ T cell count (cells/μL)	383.0 (230.3–659.9)	279.8 (126.6–564.0)	0.03
CD4^+^/CD8^+^ ratio	0.10 (0.04–0.18)	0.06 (0.03–0.15)	0.06
HIV RNA (log_10_ copies/mL)	5.51 (4.84–6.00)	5.58 (5.02–5.89)	0.62
WBC (×10^9^/L)	5.13 (3.59–6.90)	5.95 (4.39–8.72)	0.10
Neutrophils (%)	74.8 (63.6–83.0)	81.4 (71.9–92.1)	<0.01
CRP (mg/L)	40.09 (8.54–74.53)	22.07 (7.85–51.89)	0.26
PCT (ng/mL)	0.10 (0.05–0.40)	0.10 (0.04–0.24)	0.34
Albumin (g/L)	33.4 ± 6.5	30.6 ± 5.6	0.01
eGFR (mL/min/1.73 m^2^)	115.9 (91.9–140.2)	141.8 (104.8–169.7)	0.02
*Pneumocystis jirovecii* (PCP), *n* (%)	86 (54.8)	26 (81.3)	<0.01
Mycobacteria, *n* (%)	46 (29.3)	9 (28.1)	0.89
FPI, *n* (%)	19 (12.1)	9 (28.1)	0.16
Clinical Outcomes			
Mechanical ventilation, *n* (%)	18 (11.5)	11 (34.4)	<0.01
Length of hospital stay (days)	17.0 (11.0–24.5)	30.5 (20.0–49.0)	<0.001
90-day all-cause mortality, *n* (%)	14 (8.9)	7 (21.9)	0.06

Abbreviations: ART, Antiretroviral therapy; CMV, *Cytomegalovirus*; CRP, C-reactive protein; eGFR, Estimated glomerular filtration rate; HBV, Hepatitis B virus; HIV, Human immunodeficiency virus; PCP, *Pneumocystis jirovecii* pneumonia; PCT, Procalcitonin; WBC, White blood cell count; FPI, fungal pulmonary infections, including aspergillosis, *Talaromyces marneffei* infection, and cryptococcosis.

**Table 4:** Impact of BALF CMV DNA load and anti-CMV therapy on 90-day mortality.

Group	Number of Patients (*n*)	90-Day Mortality, *n* (%)	*p* Value
Low CMV/Untreated	134	11 (8.21%)	Ref.
Low CMV/Treated	23	3 (13.04%)	0.434
High CMV/Untreated	19	4 (21.05%)	0.095
High CMV/Treated	13	3 (23.07%)	0.111

Abbreviations: *p*-values represent the comparison of 90-day mortality between each respective group and the “Low CMV/Untreated” reference group (Ref.). CMV, *Cytomegalovirus*.

## Data Availability

The data that support the findings of this study are not publicly available because they contain information that could compromise the privacy of research participants. However, anonymized data are available from the corresponding author upon reasonable request.
